# Mutual Admiration Societies

**Published:** 1897-09-18

**Authors:** 


### Medical Progress and Hospital Clinics.

[*The Editor will be glad to receive offers of co-operation and contributions from members of the profession. All letters should be addressed to* The Editor, at the Office, 28 & 29, Southampton Street, Strand, London, W.C.]

### MUTUAL ADMIRATION SOCIETIES.

In endeavouring to explain the great activity in operative surgery which is so characteristic of the present day, Mr. Christopher Heath mentions as a contributory cause "the enormously increased opportunity for the publication of a success at one of the numerous mutual admiration societies," &c. We think this remark is unfair—and unfair in a double sense, first, as suggesting that praise rather than criticism is sought at the meetings of our medical societies, and next as hinting that their function is the publication of success. No one can attend the societies regularly without quickly becoming aware that "mutual admiration" is the very last thing of which they are guilty. Nor would it be correct to think that successes alone are recorded at their meetings. The very opposite is the case. The societies in London offer a fair, although sometimes a rather dull transcript of modern medical and surgical progress. Good work is no doubt appreciated, as indeed it should be; but the best of work will meet with criticism, while in case of too great exaltation is there not always the array of "results" at the Pathological to pull down pride of heart?

